# 7-(4-Meth­oxy­phen­yl)-4,9-dimethyl-*N*-(4-methyl­phen­yl)-5,12-diaza­tetra­phen-6-amine

**DOI:** 10.1107/S1600536811006209

**Published:** 2011-03-02

**Authors:** K. N. Vennila, K. Prabha, K. J. Rajendra Prasad, D. Velmurugan

**Affiliations:** a Centre of Advanced Study in Crystallography and Biophysics, University of Madras, Guindy Campus, Chennai 600 025, India; b Department of Chemistry, Bharathiar University, Coimbatore 641 046, India

## Abstract

In the title compound, C_32_H_27_N_3_O, the fused tetra­cycilc ring system is essentially planar [r.m.s. deviation = 0.07 (7) Å]. An intra­molecular N—H⋯π(arene) inter­action and a weak intra­molecular C—H⋯N hydrogen bond may influence the mol­ecular conformation. In the crystal, weak inter­molecular C—H⋯N hydrogen bonds link the mol­ecules into centrosymmetric dimers, forming *R*_2_^2^(14) motifs. In addition, weak π–π stacking inter­actions with centroid–centroid distances in the range 3.578 (1)–3.739 (1) Å provide further stabilization.

## Related literature

For the biological activity of naphthyridine derivatives, see: Gopalsamy *et al.* (2007 ▶); Kim *et al.* (2009 ▶); Nittoli *et al.* (2010 ▶); Bedard *et al.* (2000 ▶). For the structures of related naphthrydine derivatives, see: Peng *et al.* (2009 ▶); Seebacher *et al* . (2010) ▶; Vennila *et al.* (2010*a* ▶,*b* ▶). For standard bond lengths, see: Allen *et al.* (1987 ▶). For hydrogen-bond motifs, see: Bernstein *et al.* (1995 ▶).
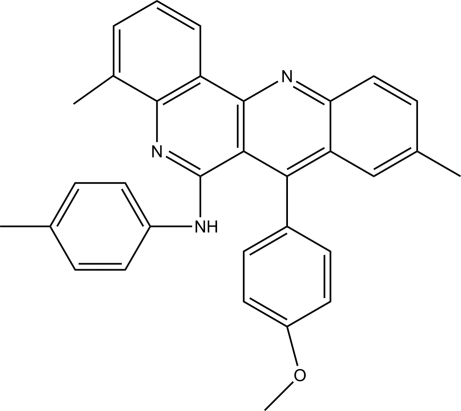


## Experimental

### 

#### Crystal data

C_32_H_27_N_3_O
*M* *_r_* = 469.57
Monoclinic, 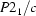

*a* = 8.3816 (6) Å
*b* = 23.1651 (13) Å
*c* = 12.8548 (7) Å
β = 91.171 (3)°
*V* = 2495.4 (3) Å^3^
*Z* = 4
Mo *K*α radiation
μ = 0.08 mm^−1^
*T* = 293 K
0.29 × 0.24 × 0.23 mm

#### Data collection

Bruker SMART APEXII area-detector diffractometer
Absorption correction: multi-scan (*SADABS*; Bruker, 2004) *T*_min_ = 0.978, *T*_max_ = 0.983
24206 measured reflections
6239 independent reflections
3904 reflections with *I* > 2σ(*I*)
*R*_int_ = 0.029

#### Refinement

*R*[*F* ^2^ > 2σ(*F* ^2^)] = 0.053
*wR*(*F* ^2^) = 0.181
*S* = 0.95
6239 reflections
329 parameters
H-atom parameters constrained
Δρ_max_ = 0.27 e Å^−3^
Δρ_min_ = −0.23 e Å^−3^

### 

Data collection: *APEX2* (Bruker, 2007 ▶); cell refinement: *SAINT* (Bruker, 2007 ▶); data reduction: *SAINT*; program(s) used to solve structure: *SHELXS97* (Sheldrick, 2008 ▶); program(s) used to refine structure: *SHELXL97* (Sheldrick, 2008 ▶); molecular graphics: *ORTEP-3* (Farrugia, 1997 ▶); software used to prepare material for publication: *SHELXL97* and *PLATON* (Spek, 2009 ▶).

## Supplementary Material

Crystal structure: contains datablocks global, I. DOI: 10.1107/S1600536811006209/lh5193sup1.cif

Structure factors: contains datablocks I. DOI: 10.1107/S1600536811006209/lh5193Isup2.hkl

Additional supplementary materials:  crystallographic information; 3D view; checkCIF report

## Figures and Tables

**Table 1:** Hydrogen-bond geometry (Å, °) *Cg* is the centroid of the C17–C22 ring.

*D*—H⋯*A*	*D*—H	H⋯*A*	*D*⋯*A*	*D*—H⋯*A*
N3—H3⋯*Cg*	0.86	2.48	3.336 (3)	176
C28—H28⋯N1	0.93	2.37	2.927 (3)	118
C18—H18⋯N2^i^	0.93	2.55	3.435 (2)	159

Symmetry code: (i) 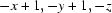.

**Fig. 1.**
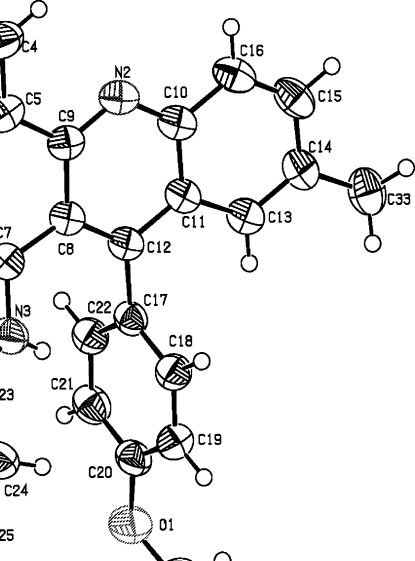
The molecular structure of the title compound, showing thermal ellipsoids drawn at 50% probability level.

**Fig. 2.**
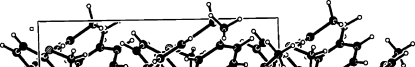
The crystal packing of the title compound with hydrogen bonds shown as dashed lines.

## supplementary crystallographic information

### Comment

Dibenzo-naphthyridine analogs have been reported to be good Phosphoinositide-Dependent Kinase (PDK-1) inhibitors. Gopalsamy *et al.* (2007) and Kim *et al.* (2009) have described the synthesis and structure activity relationship analysis of a novel series of benzo[c][2,7]naphthyridines as potent PDK-1 inhibitors. Recently a few X-ray crystal structures of PDK-1 and dibenzo[2,7] naphthyridine analog complexes have been reported (Gopalsamy *et al.*, 2007; Nittoli *et al.*, 2010). A series of dibenzo-naphthyridines were sucessfully tested for anticancer assays (Gopalsamy *et al.*, 2007; Nittoli *et al.*, 2010). The naphthyridine compounds were also proven to exhibit potent activity against human cytomegalovirus (Bedard *et al.* , 2000). As we are focussing on heterocyclic naphthyridine derivatives with potential biological properties, the crystal structure of the title compound was determined.

The molecular structure of the title compound is shown in Fig. 1. The bond lengths and angles are in the normal ranges (Allen  *et al.*, 1987). The fused tetracyclic ring system is essentially planar in geometry as was previously reported for a related compounds (Vennila *et al.*, 2010, 2011; Seebacher *et al.* 2010; Peng  *et al.*  2009). An intramolecular N—H···π(arene) interaction and a weak intramolecular C—H···N hydrogen bond may influence the molecular conformation. In the crystal, weak intermolecular C—H···N hydrogen bonds link the molecules into centrosymmetric dimers forming R_2_^2^(14) motifs (Bernstein *et al.*, 1995) (see Fig. 2). In addition, weak π–π stacking interactions with centroid to centroid distances in the range 3.578 (1) - 3.739 (1) Å provide additional stabilization.

### Experimental

A mixture of 4',4''-dimethyl-2,4-bis-(*N*-phenylamino) quinoline (0.0010 mol) and *p*-methoxybenzoic acid (0.0011 mol) was added to polyphosphoric acid (3 g of P_2_O_5_ in 1.5 mL of H_3_PO_4_) and kept at 323-328K for 5 h. The reaction was monitored by TLC. After the completion of the reaction, the reaction mixture was poured into ice water and neutralised with saturated NaHCO_3_ solution to remove the excess of *p*-methoxy benzoic acid. The precipitate was filtered, dried and purified by column chromatography over silica gel using petroleum ether : ethyl acetate (98 : 2). The product was recrystallised using ethyl acetate.

### Refinement

The H-atoms were positioned geometrically and treated as riding atoms: C—H =0.93 Å H-aromatic, C—H = 0.96 Å H-methyl, and N—H = 0.86 Å, with *U*_iso_ = k×*U*_eq_(parent C or N-atom), where k = 1.5 for methyl H-atoms, and = 1.2 for all other H-atoms.

### Crystal data

**Table:**

C_32_H_27_N_3_O	*F*(000) = 992
*M**_r_* = 469.57	*D*_x_ = 1.250 Mg m^−^^3^
Monoclinic, *P*2_1_/*c*	Mo *K*α radiation, λ = 0.71073 Å
Hall symbol: -P 2ybc	Cell parameters from 6327 reflections
*a* = 8.3816 (6) Å	θ = 1.8–28.5°
*b* = 23.1651 (13) Å	µ = 0.08 mm^−^^1^
*c* = 12.8548 (7) Å	*T* = 293 K
β = 91.171 (3)°	Block, yellow
*V* = 2495.4 (3) Å^3^	0.29 × 0.24 × 0.23 mm
*Z* = 4	

### Data collection

**Table:**

Bruker SMART APEXII area-detector diffractometer	6239 independent reflections
Radiation source: fine-focus sealed tube	3904 reflections with *I* > 2σ(*I*)
graphite	*R*_int_ = 0.029
ω and φ scans	θ_max_ = 28.5°, θ_min_ = 1.8°
Absorption correction: multi-scan (*SADABS*; Bruker, 2004)	*h* = −11→11
*T*_min_ = 0.978, *T*_max_ = 0.983	*k* = −29→30
24206 measured reflections	*l* = −17→16

### Refinement

**Table:**

Refinement on *F*^2^	Primary atom site location: structure-invariant direct methods
Least-squares matrix: full	Secondary atom site location: difference Fourier map
*R*[*F*^2^ > 2σ(*F*^2^)] = 0.053	Hydrogen site location: inferred from neighbouring sites
*wR*(*F*^2^) = 0.181	H-atom parameters constrained
*S* = 0.95	*w* = 1/[σ^2^(*F*_o_^2^) + (0.0992*P*)^2^ + 0.5542*P*] where *P* = (*F*_o_^2^ + 2*F*_c_^2^)/3
6239 reflections	(Δ/σ)_max_ = 0.001
329 parameters	Δρ_max_ = 0.27 e Å^−^^3^
0 restraints	Δρ_min_ = −0.23 e Å^−^^3^

### Special details

**Table:**

Geometry. All esds (except the esd in the dihedral angle between two l.s. planes) are estimated using the full covariance matrix. The cell esds are taken into account individually in the estimation of esds in distances, angles and torsion angles; correlations between esds in cell parameters are only used when they are defined by crystal symmetry. An approximate (isotropic) treatment of cell esds is used for estimating esds involving l.s. planes.
Refinement. Refinement of F^2^ against ALL reflections. The weighted R-factor wR and goodness of fit S are based on F^2^, conventional R-factors R are based on F, with F set to zero for negative F^2^. The threshold expression of F^2^ > 2sigma(F^2^) is used only for calculating R-factors(gt) etc. and is not relevant to the choice of reflections for refinement. R-factors based on F^2^ are statistically about twice as large as those based on F, and R- factors based on ALL data will be even larger.

### Fractional atomic coordinates and isotropic or equivalent isotropic displacement parameters (Å^2^)

**Table:**

	*x*	*y*	*z*	*U*_iso_*/*U*_eq_	
C11	0.27001 (18)	0.47982 (7)	0.01559 (12)	0.0415 (4)	
N2	0.38310 (17)	0.54757 (6)	0.14162 (11)	0.0481 (3)	
C9	0.44758 (19)	0.50366 (7)	0.19420 (12)	0.0437 (4)	
C8	0.43129 (19)	0.44464 (7)	0.16148 (12)	0.0419 (4)	
C17	0.29497 (18)	0.37335 (7)	0.03664 (12)	0.0416 (4)	
C6	0.6097 (2)	0.47172 (8)	0.34423 (13)	0.0506 (4)	
C10	0.29963 (19)	0.53681 (7)	0.05320 (13)	0.0452 (4)	
C12	0.33652 (18)	0.43309 (6)	0.07278 (12)	0.0411 (4)	
C7	0.52499 (19)	0.40261 (7)	0.22420 (12)	0.0445 (4)	
C14	0.11930 (19)	0.51950 (8)	−0.13214 (13)	0.0488 (4)	
N1	0.60278 (17)	0.41521 (6)	0.30973 (11)	0.0509 (4)	
C13	0.1761 (2)	0.47325 (7)	−0.07674 (13)	0.0471 (4)	
H13	0.1524	0.4362	−0.1004	0.056*	
C5	0.5387 (2)	0.51709 (7)	0.28813 (13)	0.0508 (4)	
N3	0.53552 (18)	0.34709 (6)	0.18772 (12)	0.0527 (4)	
H3	0.4677	0.3388	0.1389	0.063*	
C22	0.1889 (2)	0.34023 (7)	0.09339 (13)	0.0467 (4)	
H22	0.1425	0.3560	0.1520	0.056*	
O1	0.1768 (2)	0.20499 (5)	−0.04304 (11)	0.0739 (4)	
C20	0.2205 (2)	0.26068 (7)	−0.02185 (14)	0.0523 (4)	
C16	0.2387 (2)	0.58410 (7)	−0.00458 (15)	0.0534 (4)	
H16	0.2565	0.6215	0.0194	0.064*	
C19	0.3250 (2)	0.29293 (7)	−0.08088 (14)	0.0548 (5)	
H19	0.3705	0.2770	−0.1397	0.066*	
C18	0.3608 (2)	0.34901 (7)	−0.05119 (13)	0.0493 (4)	
H18	0.4302	0.3707	−0.0909	0.059*	
C15	0.1548 (2)	0.57559 (8)	−0.09457 (16)	0.0551 (5)	
H15	0.1195	0.6074	−0.1326	0.066*	
C23	0.6376 (2)	0.30140 (7)	0.21616 (14)	0.0517 (4)	
C1	0.6987 (3)	0.48302 (9)	0.43698 (15)	0.0646 (5)	
C21	0.1515 (2)	0.28481 (7)	0.06463 (14)	0.0529 (4)	
H21	0.0798	0.2635	0.1033	0.063*	
C33	0.0213 (2)	0.51229 (9)	−0.23006 (15)	0.0622 (5)	
H33A	0.0461	0.4759	−0.2616	0.093*	
H33B	0.0449	0.5430	−0.2774	0.093*	
H33C	−0.0900	0.5133	−0.2139	0.093*	
C4	0.5588 (3)	0.57407 (9)	0.32314 (16)	0.0680 (6)	
H4	0.5125	0.6045	0.2861	0.082*	
C26	0.8354 (2)	0.20560 (8)	0.25512 (18)	0.0661 (5)	
C28	0.7225 (3)	0.29651 (9)	0.30897 (17)	0.0732 (6)	
H28	0.7150	0.3250	0.3596	0.088*	
C24	0.6517 (3)	0.25781 (8)	0.14459 (18)	0.0736 (6)	
H24	0.5944	0.2600	0.0820	0.088*	
C25	0.7489 (3)	0.21103 (9)	0.1638 (2)	0.0809 (7)	
H25	0.7562	0.1823	0.1137	0.097*	
C27	0.8194 (3)	0.24844 (10)	0.3259 (2)	0.0813 (7)	
H27	0.8757	0.2456	0.3888	0.098*	
C29	0.7759 (3)	0.43458 (11)	0.49655 (17)	0.0853 (7)	
H29A	0.8283	0.4496	0.5579	0.128*	
H29B	0.8529	0.4159	0.4537	0.128*	
H29C	0.6961	0.4072	0.5162	0.128*	
C2	0.7139 (3)	0.53922 (11)	0.46798 (18)	0.0831 (7)	
H2	0.7713	0.5473	0.5289	0.100*	
C30	0.9433 (3)	0.15394 (10)	0.2742 (2)	0.0919 (8)	
H30A	1.0215	0.1521	0.2208	0.138*	
H30B	0.8806	0.1193	0.2731	0.138*	
H30C	0.9961	0.1578	0.3408	0.138*	
C3	0.6470 (3)	0.58475 (11)	0.41220 (19)	0.0859 (7)	
H3A	0.6619	0.6225	0.4351	0.103*	
C32	0.2335 (5)	0.18004 (10)	−0.13340 (19)	0.1118 (11)	
H32A	0.2088	0.2046	−0.1917	0.168*	
H32B	0.1839	0.1431	−0.1439	0.168*	
H32C	0.3470	0.1752	−0.1271	0.168*	

### Atomic displacement parameters (Å^2^)

**Table:**

	*U*^11^	*U*^22^	*U*^33^	*U*^12^	*U*^13^	*U*^23^
C11	0.0348 (8)	0.0400 (8)	0.0498 (8)	−0.0001 (6)	0.0044 (7)	0.0023 (6)
N2	0.0441 (8)	0.0410 (7)	0.0595 (8)	−0.0014 (6)	0.0068 (7)	−0.0046 (6)
C9	0.0387 (8)	0.0436 (8)	0.0490 (9)	−0.0023 (7)	0.0078 (7)	−0.0036 (7)
C8	0.0372 (8)	0.0406 (8)	0.0480 (8)	−0.0009 (6)	0.0036 (7)	−0.0003 (6)
C17	0.0391 (8)	0.0385 (8)	0.0467 (8)	0.0003 (6)	−0.0060 (7)	0.0025 (6)
C6	0.0475 (10)	0.0572 (10)	0.0473 (9)	−0.0041 (8)	0.0044 (8)	−0.0061 (7)
C10	0.0363 (8)	0.0414 (8)	0.0582 (10)	−0.0002 (7)	0.0083 (7)	0.0000 (7)
C12	0.0359 (8)	0.0388 (8)	0.0488 (8)	0.0003 (6)	0.0045 (7)	0.0000 (6)
C7	0.0388 (8)	0.0462 (9)	0.0484 (9)	−0.0013 (7)	0.0007 (7)	0.0010 (7)
C14	0.0356 (8)	0.0539 (10)	0.0571 (10)	0.0041 (7)	0.0052 (7)	0.0107 (7)
N1	0.0479 (8)	0.0564 (9)	0.0483 (8)	−0.0009 (7)	−0.0005 (7)	−0.0016 (6)
C13	0.0409 (9)	0.0447 (9)	0.0557 (9)	0.0004 (7)	0.0028 (7)	0.0011 (7)
C5	0.0494 (10)	0.0535 (10)	0.0497 (9)	−0.0064 (8)	0.0061 (8)	−0.0097 (7)
N3	0.0519 (9)	0.0453 (8)	0.0603 (9)	0.0034 (6)	−0.0162 (7)	−0.0024 (6)
C22	0.0419 (9)	0.0458 (9)	0.0522 (9)	0.0014 (7)	−0.0011 (7)	0.0018 (7)
O1	0.1046 (12)	0.0424 (7)	0.0739 (9)	−0.0111 (7)	−0.0147 (8)	−0.0056 (6)
C20	0.0616 (11)	0.0377 (8)	0.0567 (10)	−0.0031 (7)	−0.0163 (8)	0.0017 (7)
C16	0.0444 (9)	0.0409 (9)	0.0751 (12)	0.0041 (7)	0.0066 (9)	0.0035 (8)
C19	0.0659 (12)	0.0489 (10)	0.0494 (9)	0.0032 (8)	−0.0044 (8)	−0.0061 (7)
C18	0.0534 (10)	0.0451 (9)	0.0496 (9)	−0.0033 (7)	0.0024 (8)	0.0002 (7)
C15	0.0403 (9)	0.0505 (10)	0.0746 (12)	0.0077 (8)	0.0063 (9)	0.0160 (8)
C23	0.0469 (10)	0.0455 (9)	0.0623 (10)	0.0013 (7)	−0.0075 (8)	0.0051 (8)
C1	0.0648 (13)	0.0780 (14)	0.0509 (10)	−0.0048 (10)	−0.0003 (9)	−0.0100 (9)
C21	0.0513 (10)	0.0452 (9)	0.0621 (10)	−0.0072 (8)	−0.0033 (8)	0.0074 (8)
C33	0.0490 (11)	0.0744 (13)	0.0630 (12)	0.0031 (9)	−0.0017 (9)	0.0145 (9)
C4	0.0821 (15)	0.0560 (11)	0.0660 (12)	−0.0063 (10)	0.0021 (11)	−0.0150 (9)
C26	0.0553 (12)	0.0505 (11)	0.0919 (15)	0.0047 (9)	−0.0111 (11)	0.0118 (10)
C28	0.0827 (15)	0.0653 (13)	0.0705 (13)	0.0191 (11)	−0.0235 (11)	−0.0045 (10)
C24	0.0892 (16)	0.0523 (11)	0.0778 (13)	0.0153 (10)	−0.0311 (12)	−0.0069 (10)
C25	0.0951 (18)	0.0512 (12)	0.0953 (16)	0.0183 (11)	−0.0236 (14)	−0.0100 (11)
C27	0.0821 (16)	0.0747 (14)	0.0857 (15)	0.0190 (12)	−0.0317 (13)	0.0073 (12)
C29	0.0919 (18)	0.1044 (19)	0.0587 (12)	0.0039 (14)	−0.0180 (12)	−0.0034 (12)
C2	0.0952 (19)	0.0912 (18)	0.0624 (13)	−0.0107 (14)	−0.0125 (12)	−0.0218 (12)
C30	0.0839 (17)	0.0659 (14)	0.125 (2)	0.0222 (12)	−0.0138 (15)	0.0159 (13)
C3	0.109 (2)	0.0742 (15)	0.0737 (14)	−0.0156 (14)	−0.0092 (14)	−0.0303 (12)
C32	0.209 (4)	0.0567 (14)	0.0696 (14)	−0.0158 (17)	−0.0010 (18)	−0.0162 (11)

### Geometric parameters (Å, °)

**Table:**

C11—C12	1.416 (2)	C19—H19	0.9300
C11—C13	1.419 (2)	C18—H18	0.9300
C11—C10	1.426 (2)	C15—H15	0.9300
N2—C9	1.330 (2)	C23—C24	1.373 (3)
N2—C10	1.346 (2)	C23—C28	1.381 (3)
C9—C8	1.436 (2)	C1—C2	1.367 (3)
C9—C5	1.449 (2)	C1—C29	1.498 (3)
C8—C12	1.402 (2)	C21—H21	0.9300
C8—C7	1.479 (2)	C33—H33A	0.9600
C17—C18	1.386 (2)	C33—H33B	0.9600
C17—C22	1.392 (2)	C33—H33C	0.9600
C17—C12	1.498 (2)	C4—C3	1.372 (3)
C6—N1	1.383 (2)	C4—H4	0.9300
C6—C5	1.400 (3)	C26—C27	1.355 (3)
C6—C1	1.418 (3)	C26—C25	1.373 (3)
C10—C16	1.413 (2)	C26—C30	1.517 (3)
C7—N1	1.300 (2)	C28—C27	1.393 (3)
C7—N3	1.372 (2)	C28—H28	0.9300
C14—C13	1.367 (2)	C24—C25	1.375 (3)
C14—C15	1.416 (3)	C24—H24	0.9300
C14—C33	1.498 (3)	C25—H25	0.9300
C13—H13	0.9300	C27—H27	0.9300
C5—C4	1.403 (2)	C29—H29A	0.9600
N3—C23	1.405 (2)	C29—H29B	0.9600
N3—H3	0.8600	C29—H29C	0.9600
C22—C21	1.371 (2)	C2—C3	1.387 (4)
C22—H22	0.9300	C2—H2	0.9300
O1—C20	1.367 (2)	C30—H30A	0.9600
O1—C32	1.390 (3)	C30—H30B	0.9600
C20—C19	1.388 (3)	C30—H30C	0.9600
C20—C21	1.381 (3)	C3—H3A	0.9300
C16—C15	1.356 (3)	C32—H32A	0.9600
C16—H16	0.9300	C32—H32B	0.9600
C19—C18	1.385 (2)	C32—H32C	0.9600
			
C12—C11—C13	123.91 (14)	C24—C23—C28	117.96 (17)
C12—C11—C10	117.89 (15)	C24—C23—N3	116.10 (16)
C13—C11—C10	118.20 (14)	C28—C23—N3	125.94 (17)
C9—N2—C10	119.11 (14)	C2—C1—C6	117.7 (2)
N2—C9—C8	122.97 (15)	C2—C1—C29	121.8 (2)
N2—C9—C5	117.45 (15)	C6—C1—C29	120.41 (19)
C8—C9—C5	119.59 (15)	C22—C21—C20	119.92 (16)
C12—C8—C9	117.95 (15)	C22—C21—H21	120.0
C12—C8—C7	127.10 (14)	C20—C21—H21	120.0
C9—C8—C7	114.88 (14)	C14—C33—H33A	109.5
C18—C17—C22	118.13 (15)	C14—C33—H33B	109.5
C18—C17—C12	122.29 (14)	H33A—C33—H33B	109.5
C22—C17—C12	119.57 (14)	C14—C33—H33C	109.5
N1—C6—C5	122.05 (16)	H33A—C33—H33C	109.5
N1—C6—C1	117.53 (17)	H33B—C33—H33C	109.5
C5—C6—C1	120.34 (17)	C3—C4—C5	119.7 (2)
N2—C10—C16	118.44 (15)	C3—C4—H4	120.1
N2—C10—C11	122.79 (15)	C5—C4—H4	120.1
C16—C10—C11	118.76 (16)	C27—C26—C25	116.68 (19)
C8—C12—C11	119.12 (14)	C27—C26—C30	122.4 (2)
C8—C12—C17	123.54 (14)	C25—C26—C30	120.9 (2)
C11—C12—C17	117.28 (14)	C23—C28—C27	119.3 (2)
N1—C7—N3	117.69 (15)	C23—C28—H28	120.4
N1—C7—C8	124.38 (15)	C27—C28—H28	120.4
N3—C7—C8	117.88 (15)	C23—C24—C25	121.2 (2)
C13—C14—C15	118.26 (17)	C23—C24—H24	119.4
C13—C14—C33	121.96 (17)	C25—C24—H24	119.4
C15—C14—C33	119.78 (16)	C26—C25—C24	121.8 (2)
C7—N1—C6	120.05 (15)	C26—C25—H25	119.1
C14—C13—C11	122.22 (16)	C24—C25—H25	119.1
C14—C13—H13	118.9	C26—C27—C28	123.1 (2)
C11—C13—H13	118.9	C26—C27—H27	118.5
C6—C5—C4	119.60 (17)	C28—C27—H27	118.5
C6—C5—C9	118.62 (15)	C1—C29—H29A	109.5
C4—C5—C9	121.78 (18)	C1—C29—H29B	109.5
C7—N3—C23	131.31 (15)	H29A—C29—H29B	109.5
C7—N3—H3	114.3	C1—C29—H29C	109.5
C23—N3—H3	114.3	H29A—C29—H29C	109.5
C21—C22—C17	121.33 (16)	H29B—C29—H29C	109.5
C21—C22—H22	119.3	C1—C2—C3	122.6 (2)
C17—C22—H22	119.3	C1—C2—H2	118.7
C20—O1—C32	117.64 (18)	C3—C2—H2	118.7
O1—C20—C19	124.64 (17)	C26—C30—H30A	109.5
O1—C20—C21	115.29 (16)	C26—C30—H30B	109.5
C19—C20—C21	120.07 (15)	H30A—C30—H30B	109.5
C15—C16—C10	120.74 (16)	C26—C30—H30C	109.5
C15—C16—H16	119.6	H30A—C30—H30C	109.5
C10—C16—H16	119.6	H30B—C30—H30C	109.5
C20—C19—C18	119.30 (16)	C4—C3—C2	119.9 (2)
C20—C19—H19	120.3	C4—C3—H3A	120.0
C18—C19—H19	120.3	C2—C3—H3A	120.0
C17—C18—C19	121.22 (16)	O1—C32—H32A	109.5
C17—C18—H18	119.4	O1—C32—H32B	109.5
C19—C18—H18	119.4	H32A—C32—H32B	109.5
C16—C15—C14	121.74 (16)	O1—C32—H32C	109.5
C16—C15—H15	119.1	H32A—C32—H32C	109.5
C14—C15—H15	119.1	H32B—C32—H32C	109.5
			
C10—N2—C9—C8	−0.6 (2)	C8—C9—C5—C4	179.71 (16)
C10—N2—C9—C5	179.02 (13)	N1—C7—N3—C23	−12.1 (3)
N2—C9—C8—C12	−3.1 (2)	C8—C7—N3—C23	165.47 (16)
C5—C9—C8—C12	177.28 (13)	C18—C17—C22—C21	0.9 (2)
N2—C9—C8—C7	174.06 (14)	C12—C17—C22—C21	−178.05 (15)
C5—C9—C8—C7	−5.5 (2)	C32—O1—C20—C19	−4.0 (3)
C9—N2—C10—C16	−176.83 (14)	C32—O1—C20—C21	175.4 (2)
C9—N2—C10—C11	3.3 (2)	N2—C10—C16—C15	179.56 (15)
C12—C11—C10—N2	−2.1 (2)	C11—C10—C16—C15	−0.5 (2)
C13—C11—C10—N2	177.95 (14)	O1—C20—C19—C18	−179.70 (17)
C12—C11—C10—C16	177.95 (14)	C21—C20—C19—C18	0.9 (3)
C13—C11—C10—C16	−2.0 (2)	C22—C17—C18—C19	−1.3 (3)
C9—C8—C12—C11	4.1 (2)	C12—C17—C18—C19	177.59 (16)
C7—C8—C12—C11	−172.70 (14)	C20—C19—C18—C17	0.4 (3)
C9—C8—C12—C17	−173.20 (14)	C10—C16—C15—C14	2.5 (3)
C7—C8—C12—C17	10.0 (2)	C13—C14—C15—C16	−1.9 (2)
C13—C11—C12—C8	178.23 (14)	C33—C14—C15—C16	177.88 (16)
C10—C11—C12—C8	−1.7 (2)	C7—N3—C23—C24	−160.3 (2)
C13—C11—C12—C17	−4.3 (2)	C7—N3—C23—C28	20.4 (3)
C10—C11—C12—C17	175.80 (13)	N1—C6—C1—C2	176.14 (18)
C18—C17—C12—C8	−107.91 (19)	C5—C6—C1—C2	−0.9 (3)
C22—C17—C12—C8	71.0 (2)	N1—C6—C1—C29	−2.4 (3)
C18—C17—C12—C11	74.7 (2)	C5—C6—C1—C29	−179.42 (18)
C22—C17—C12—C11	−106.41 (17)	C17—C22—C21—C20	0.5 (3)
C12—C8—C7—N1	−175.40 (15)	O1—C20—C21—C22	179.22 (16)
C9—C8—C7—N1	7.7 (2)	C19—C20—C21—C22	−1.4 (3)
C12—C8—C7—N3	7.2 (2)	C6—C5—C4—C3	−0.3 (3)
C9—C8—C7—N3	−169.75 (14)	C9—C5—C4—C3	−179.60 (19)
N3—C7—N1—C6	173.38 (15)	C24—C23—C28—C27	0.7 (3)
C8—C7—N1—C6	−4.1 (2)	N3—C23—C28—C27	−180.0 (2)
C5—C6—N1—C7	−1.9 (2)	C28—C23—C24—C25	−0.9 (3)
C1—C6—N1—C7	−178.88 (16)	N3—C23—C24—C25	179.7 (2)
C15—C14—C13—C11	−0.7 (2)	C27—C26—C25—C24	0.5 (4)
C33—C14—C13—C11	179.49 (14)	C30—C26—C25—C24	−179.3 (2)
C12—C11—C13—C14	−177.29 (15)	C23—C24—C25—C26	0.3 (4)
C10—C11—C13—C14	2.6 (2)	C25—C26—C27—C28	−0.7 (4)
N1—C6—C5—C4	−175.65 (17)	C30—C26—C27—C28	179.1 (2)
C1—C6—C5—C4	1.2 (3)	C23—C28—C27—C26	0.1 (4)
N1—C6—C5—C9	3.7 (2)	C6—C1—C2—C3	−0.4 (4)
C1—C6—C5—C9	−179.45 (15)	C29—C1—C2—C3	178.1 (2)
N2—C9—C5—C6	−179.17 (14)	C5—C4—C3—C2	−0.9 (4)
C8—C9—C5—C6	0.4 (2)	C1—C2—C3—C4	1.3 (4)
N2—C9—C5—C4	0.1 (2)		

### Hydrogen-bond geometry (Å, °)

**Table:**

Cg is the centroid of the C17–C22 ring.

**Table:**

*D*—H···*A*	*D*—H	H···*A*	*D*···*A*	*D*—H···*A*
N3—H3···Cg	0.86	2.48	3.336 (3)	176
C28—H28···N1	0.93	2.37	2.927 (3)	118
C18—H18···N2^i^	0.93	2.55	3.435 (2)	159

Symmetry codes:  (i) −*x*+1, −*y*+1, −*z*.
